# Application of eHealth Tools in Anticoagulation Management After Cardiac Valve Replacement: Scoping Review Coupled With Bibliometric Analysis

**DOI:** 10.2196/48716

**Published:** 2024-01-05

**Authors:** Ying Wu, Xiaohui Wang, Mengyao Zhou, Zhuoer Huang, Lijuan Liu, Li Cong

**Affiliations:** 1 Center for Moral Culture Hunan Normal University Changsha China; 2 School of Medicine Hunan Normal University Changsha China; 3 Teaching and Research Section of Clinical Nursing Xiangya Hospital of Central South University Changsha China

**Keywords:** eHealth tool, cardiac valve replacement, anticoagulation management, scoping review, bibliometrics analysis, rehabilitation

## Abstract

**Background:**

Anticoagulation management can effectively prevent complications in patients undergoing cardiac valve replacement (CVR). The emergence of eHealth tools provides new prospects for the management of long-term anticoagulants. However, there is no comprehensive summary of the application of eHealth tools in anticoagulation management after CVR.

**Objective:**

Our objective is to clarify the current state, trends, benefits, and challenges of using eHealth tools in the anticoagulation management of patients after CVR and provide future directions and recommendations for development in this field.

**Methods:**

This scoping review follows the 5-step framework developed by Arksey and O’Malley. We searched 5 databases such as PubMed, MEDLINE, Web of Science, CINAHL, and Embase using keywords such as “eHealth,” “anticoagulation,” and “valve replacement.” We included papers on the practical application of eHealth tools and excluded papers describing the underlying mechanisms for developing eHealth tools. The search time ranged from the database inception to March 1, 2023. The study findings were reported according to the PRISMA-ScR (Preferred Reporting Items for Systematic Reviews and Meta-Analyses extension for Scoping Reviews). Additionally, VOSviewer (version 1.6.18) was used to construct visualization maps of countries, institutions, authors, and keywords to investigate the internal relations of included literature and to explore research hotspots and frontiers.

**Results:**

This study included 25 studies that fulfilled the criteria. There were 27,050 participants in total, with the sample size of the included studies ranging from 49 to 13,219. The eHealth tools mainly include computer-based support systems, electronic health records, telemedicine platforms, and mobile apps. Compared to traditional anticoagulation management, eHealth tools can improve time in therapeutic range and life satisfaction. However, there is no significant impact observed in terms of economic benefits and anticoagulation-related complications. Bibliometric analysis suggests the potential for increased collaboration and opportunities among countries and academic institutions. Italy had the widest cooperative relationships. Machine learning and artificial intelligence are the popular research directions in anticoagulation management.

**Conclusions:**

eHealth tools exhibit promise for clinical applications in anticoagulation management after CVR, with the potential to enhance postoperative rehabilitation. Further high-quality research is needed to explore the economic benefits of eHealth tools in long-term anticoagulant therapy and the potential to reduce the occurrence of adverse events.

## Introduction

Valvular heart disease involves damage to the cardiac valves caused by various factors such as valve degeneration and rheumatic heart disease [1]. Currently, an estimated 209 million people suffer from valvular heart disease worldwide. With the aging population, it is expected that the prevalence of valvular heart disease among those aged 65 years and older will increase by 50% globally by 2030 [2,3]. Furthermore, valvular heart disease caused approximately 300,000 deaths in 2019, accounting for nearly 2% of cardiovascular disease deaths worldwide. Every year, 8.7 million life years and 10.7 million disability-adjusted life years are lost to valvular heart disease [2]. The fundamental treatment for valvular heart disease is the repair or replacement of damaged heart valves through surgery or interventional therapy. Patients need long-term oral anticoagulants after cardiac valve replacement (CVR) surgery; however, improper use of anticoagulants can lead to bleeding, embolism, eventual valve failure, repeat surgeries, and even death [4,5]. Efficient anticoagulation management (AM) plays a critical role in promoting safe medication therapy after CVR, reducing adverse events, and improving the long-term prognosis of patients.

The traditional AM model has many obstacles and challenges. On the one hand, patients need to travel between their home and hospital regularly for follow-up and laboratory examinations and long-term anticoagulant medication, which costs them a lot of time, energy, and economy after CVR and also reduces patients’ confidence in rehabilitation and treatment compliance [6]. On the other hand, it is difficult for doctors to monitor and manage patients and adjust treatment plans promptly, which may affect the quality and effect of anticoagulant therapy, and increase the risk of adverse events [7]. Therefore, the exploration of personalized, intelligent, and efficient AM models is crucial in promoting cardiac recovery among patients undergoing CVR. Several guidelines highlight the potential of eHealth tools to facilitate AM and improve medical outcomes [8] and recommend the use of eHealth tools to assist physicians with anticoagulation decision-making support [9].

eHealth tools generally refer to digital devices based on information and communication technology, which can be used for disease prevention, diagnosis, treatment, monitoring, and management [10]. eHealth tools can provide patients with convenient, efficient, and accurate medical services through digital communication and remote disease management. Some studies have found that eHealth tools may become a potentially cost-effective and effective alternative to traditional “face-to-face” anticoagulation therapy. A meta-analysis of 12 randomized controlled trials (RCTs) showed that telemedicine combined with portable coagulometers significantly improved the time in the therapeutic range (TTR) and reduced the incidence of thrombotic events in patients with cardiovascular disease [11]. Inpatients undergoing anticoagulation therapy receiving the recommended dosage of anticoagulation software can effectively reduce readmissions and 30-day mortality as well as hospitalization costs [12]. A comprehensive understanding of the application status and effectiveness of eHealth tools is essential to establish a scientific, traceable, and integrated AM model.

Although several narrative reviews and meta-analyses have summarized the usage of mobile health technology in patients with cardiovascular diseases [13-15], uncertainties remain regarding the effectiveness and current status of using eHealth tools in AM of patients after CVR. A comprehensive summary of the development, application, and interrelationships among different research papers or groups is lacking. Scoping reviews entail extensive searches and a rigorous selection of research literature on a specific topic, which can include a comprehensive overview of the current research status and trends of the topic [16]. Scientometric analysis can quantitatively evaluate cooperative relationships by statistically analyzing publications and graphically presenting the social and intellectual connections of relevant literature [17]. Through the joint analysis of scoping review and scientometric analysis, the research status and trends of a certain field can be comprehensively evaluated from different perspectives, and the blind spots and unsolved problems can be determined to provide guidance and enlightenment for further research.

By conducting a scoping review and scientometric analysis, this study aimed to (1) summarize the application status of eHealth tools in AM of patients after CVR, (2) identify the hotspots and provide guidance for future research and practice, and (3) to provide a reference for promoting the wider application and sustainable development of eHealth tools in AM of patients after CVR.

## Methods

### Design

We used Arksey and O’Malley’s [18] 5-step framework for the scoping review. This review also followed the recommended items in the PRISMA-ScR (Preferred Reporting Items for Systematic Reviews and Meta-Analyses extension for Scoping Reviews) checklist (Multimedia Appendix 1). Five commonly used databases were searched, and the papers were screened based on the title, abstract, and full text. We exported the complete records of filtered papers as plain text files and imported them into VOSviewer software (version 1.6.18; Centre for Science and Technology Studies) to build visualization maps. We chose coauthorship analysis to construct network visualization maps of countries, institutions, and authors to understand the status of research collaboration in the field of eHealth tools. By cluster analysis and keyword overlay visualization, keywords are divided into different clusters and stacked over time, which can identify different themes and current research hotspots.

### Scoping Review

In contrast to systematic and narrative reviews, scoping reviews focus on an initial appraisal of the current extent, scope, and nature of the research literature and take the dissemination process further by summarizing the relevant existing research activities. It is also an appropriate way to map the key concepts and identify knowledge gaps [19]. We aimed to provide an overview of the use of eHealth tools to assist anticoagulation therapy in patients after CVR and highlight the current status, trends, and challenges in this field. Therefore, the scoping review was appropriate for this study.

### Identifying the Research Question for Scoping Review

The first question that guided our scoping review was what are the range and effectiveness of eHealth tools services in AM of patients after CVR? The second one was what are the benefits and barriers of applying eHealth tools in AM?

### Identifying Databases and Studies

PubMed, MEDLINE, Web of Science, CINAHL, and Embase were searched from inception to March 1, 2023. Searches were not limited to a specific geographic region, and any literature published in non-English languages was excluded. Searches included combinations of free text words and index terms using Boolean operators. Moreover, a manual retrospective search of the references was conducted as a supplement. Detailed search strategies for each database are described in Multimedia Appendix 2.

### Study Selection

The Population, Concept, and Context framework is recommended by the Joanna Briggs Institute to identify the main concepts in primary review questions, guide the search strategy, and ensure application of the inclusion and exclusion criteria [20]. Therefore, we used the Population, Concept, and Context framework to regulate the scoping review process. Population was defined as patients who had undergone CVR surgery and received anticoagulant therapy postoperatively. Concept referred to the practical application and effectiveness of eHealth tools in the AM of patients after CVR. Context had no special restrictions, as eHealth tools can be applied in the patient's home, hospital, anticoagulant therapy clinic, primary care center, and so forth. The inclusion criteria were as follows: (1) publication types including cross-sectional studies, longitudinal studies, cohort studies, case-control studies, pilot studies, and RCTs; (2) published in full text; and (3) published from the inception of each database to March 1, 2023. The exclusion criteria were as follows: (1) non-English publications; (2) publication types including empirical research, reviews, editorials, reports, case reports, letters, and conference proceedings or papers or abstracts; (3) qualitative studies reporting user experiences about eHealth tools in AM of patients after CVR; and (4) studies describing only the potential mechanisms or development process of eHealth tools, rather than their practical applications. The literature records retrieved were imported into Note Express software to screen for duplicate papers. Two researchers (YW and XW) independently conducted the initial screening of the titles and abstracts based on the inclusion and exclusion criteria. Full texts were then examined for secondary screening of potentially eligible papers. Any disagreement during the screening process was resolved through discussion with the third researcher (LC), and the final selection of papers was determined based on the established criteria.

### Data Extraction and Analysis

Two researchers (YW and XW) independently extracted data from the included studies using standardized tables and cross-checking their findings. Any discrepancies were resolved through discussion with the third researcher (LC). The information extracted included authors, year, country, study design, study objective, participants’ characteristics, study location, content elements, outcome measures, and study conclusions. We summarized the literature on the services and effects of eHealth tools in the AM of patients after CVR and presented the main concepts and findings of the literature using data charts and tables.

### Bibliometric Analysis

VOSviewer is a widely used tool by researchers for bibliometric analysis, providing effective visualization and revealing connections between research materials [21]. The process of scientometric analysis in this study included 2 parts. First, coauthorship analysis was performed to explore the relationship among researchers, research institutions, and countries, which contributed to understanding the trends of scientific cooperation. We chose “coauthorship” as the analysis type, selected “countries,” “organizations,” and “authors” as the units of analysis and “full counting” as the counting method. To achieve clearer effects, the minimum number for each project was set as 1. Second, keywords network visualization and overlay visualization were analyzed to explore the hotspots and frontiers. We chose the “author keywords” in the “co-occurrence” type for analysis, the counting method was “full counting,” and the minimum cluster size to 6. We merged keywords with the same meaning and deleted redundant keywords. The minimum number was set to 1, and we obtained 40 keywords in total.

## Results

### Characteristics of the Included Studies

**Figure 1:**
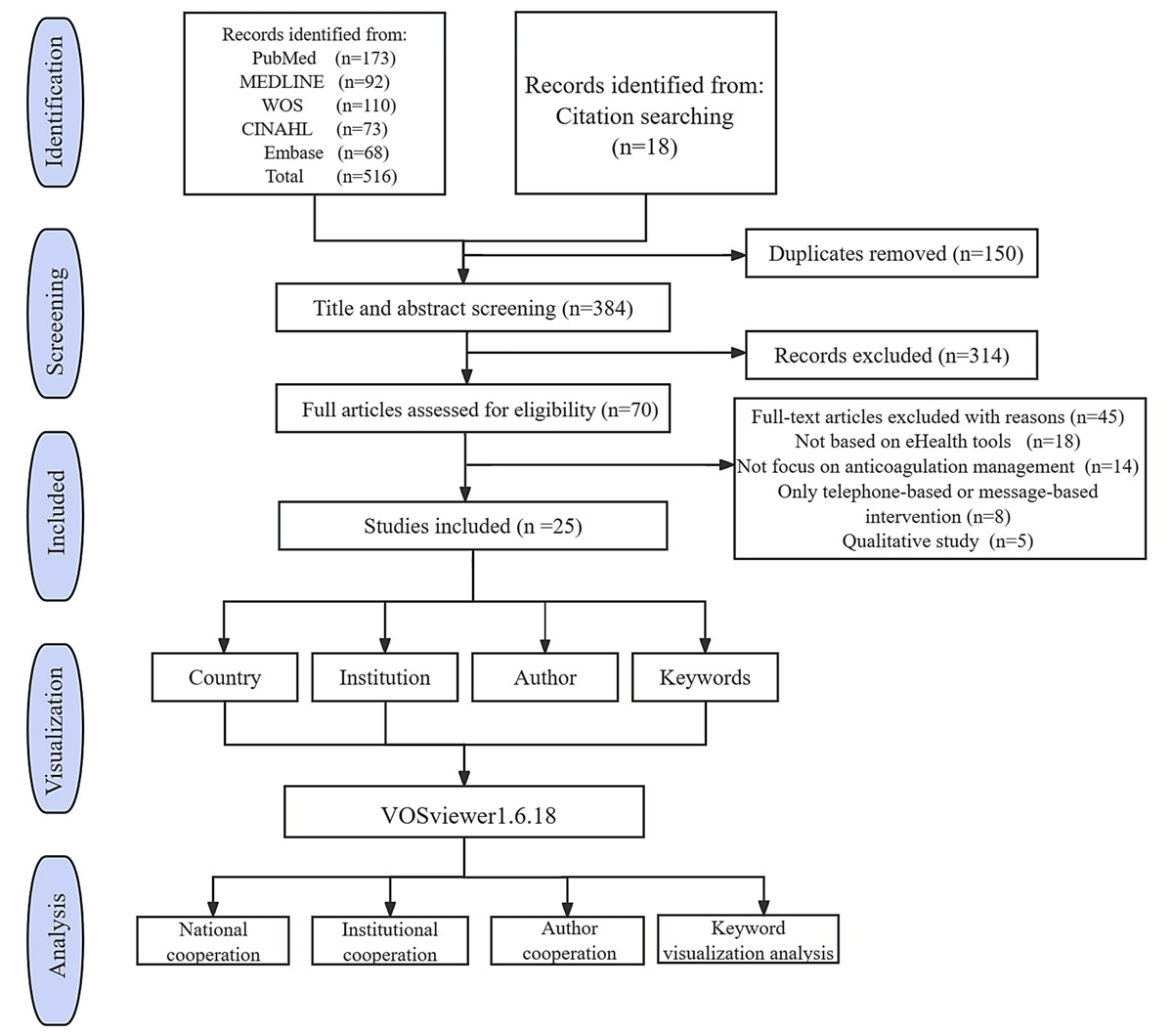
Flow diagram of the article selection process based on PRISMA-ScR (Preferred Reporting Items for Systematic Reviews and Meta-Analyses extension for Scoping Reviews) guidelines.

We conducted a systematic search of 5 databases, which yielded 534 studies that may be relevant to the topic. After eliminating duplicates, screening 384 titles and abstracts, and reviewing 70 full texts, we found that 25 papers met the inclusion and exclusion criteria. Figure 1 shows the selection process flowchart based on PRISMA-ScR [22]. The literature included in this study comprised 12 RCTs [23-34], 8 cohort studies [35-42], 2 pilot studies [43,44], 1 longitudinal study [45], 1 cross-sectional study [46], and 1 cross-over study [47]. The 25 papers comprised 27,050 participants in total, with the sample size for each study ranging from 49 to 13,219, the duration of intervention was 1-24 months, and the follow-up period was 1-514 months. Most of the participants were older than 40 years of age and had undergone mechanical valve replacement surgery. A table in Multimedia Appendix 3 provides a summary of the study characteristics and participant demographics.

### Types of Anticoagulation Services Offered by eHealth Tools

Through the comprehensive understanding and interpretation of the included literature, we found that eHealth tools can be broadly classified into 4: computer-based support systems, electronic health records (EHRs), telemedicine platforms, and mobile apps. The application scenarios of eHealth tools are gradually expanding from large general hospitals to primary health care centers and finally to patients’ homes. Most studies have reported the beneficial effects of eHealth tools on the rehabilitation outcome and anticoagulation quality in patients undergoing CVR, reflecting the potential and application prospect of eHealth tools in this field. Table 1 shows the specific types of eHealth tools and their application scenarios. Multimedia Appendix 3 shows the specific services provided by eHealth tools and their impacts on anticoagulation outcomes.

**Table 1:** Types and application scenarios of eHealth tools (N=25).

Author, Year, Country	eHealth tools	Places
Fitzmaurice et al (1996) [23], UK	Decision support systems (Anticoagulation Management Support System, Warwick)	Primary care clinic
Fitzmaurice et al (2000) [25], UK	Softop Information (Warwick, UK)	Primary care practices
Testa et al. (2006) [44], Italy	Electronic patient records (TaoNet, EDP-Progetti, Bolzano, Italy)	Peripheral health units
Poller et al (2008) [27], UK	PARMA5^a^ (Instrumentation Laboratory, Milan, Italy) and DAWN AC (program 4S Dawn Clinical Software, Milnthorpe, UK)	Hospital
Ryan et al (2009) [28], Ireland	CoagCare (ZyCare Inc., Chapel Hill, NC, United States)	Home
Cafolla et al (2011) [39], Italy	Automated computer systems coagulometer (HemoSense, San Jose, CA, United States)	Hospital
Bussey et al (2013) [43], United States	ClotFree system (ClotFree; Genesis Advanced Technologies, Lakehills, TX, United States)	Clinical research center
Ferrando et al (2015) [40], Spain	SintromacWebInternet-based system (Grifols, Barcelona, Spain)	Home
Cao et al (2018) [37], China	Digital anticoagulation clinic	Home and hospital
Zhu et al (2021) [32], China	A mobile user interface medical network follow-up platform	Home
Cao et al (2021) [42], China	Alfalfa	Home
Alanazi et al (2022) [47], Saudi Arabia	WhatsApp	Home
Amruthlal et al (2022) [46], India	Smartphone app	Home
Ageno and Turpie (1998) [24], Canada	DAWN AC (Business Technology, Milnthorpe, Cumbria, the UK)	Hospital
Manotti et al (2001) [26], Italy	PARMA system (release 3.2, Parma, Italy)	Manotti et al (2001), Italy
O'Shea et al (2008) [35], United States	Management program (CoagCare, ZyCare Inc, Chapel Hill, NC, United States)	Home
Soliman Hamad et al (2009) [29], the Netherlands	Anticoagulant aid website	Home
Christensen et al (2011) [30], Denmark	Computer system (CSO/AC; IntraMed A/S, Værløse, Denmark)	Home
Thompson et al (2013) [33], United States	Automated computer systems coagulometer (HemoSense, San Jose, CA, United States)	Hospital
Koertke et al (2015) [34], Germany	The Institute for Applied Telemedicine	Heart centers
Talboom et al (2017) [38], the Netherlands	Portavita eHealth platform	Home
Brasen et al (2019) [31], Denmark	Computer system (CSO/AC; IntraMed A/S, Værløse, Denmark)	Home
Jiang et al (2021) [41], China	Yixing App	Home
Jiang et al (2022) [36], China	Alfalfa	Home
Erba et al (2022) [45], Italy	PARMA GTS (Werfen, Milan, Italy) and WhatsApp	Anticoagulant clinic and home

^a^PARMA: Program for Archive, Refertation, and Monitoring of Anticoagulated patients.

Of the 25 included studies, 6 used computer-based support systems [23-27,39], 4 used EHR [33,38,43,44], 9 applied telemedicine platforms [28-32,34,35,37,40], and 6 used mobile apps [36,41,45-47]. Computer-based support systems are computer applications designed to aid clinicians in making diagnostic and therapeutic decisions in patient care [48]. Such support systems are typically installed in hospitals, large clinics for anticoagulant therapy, and primary health care centers. They can help doctors predict the appropriate dose of anticoagulants and the timing of the next international normalized ratio (INR) test based on the patient’s latest INR value [23-27,39]. EHRs are generated by 1 or more interactions between medical institutions and patients [49]. Doctors use these tools to record the patients’ medication information, laboratory measurement data, clinical history, or symptoms of discomfort for reference in the next visit. They use this information to make the best decision possible and summarize their clinical experience, which provides an important basis for improving the overall medical level. EHR can be shared between primary health care clinics and hospitals through a central database to support CVR surgical follow-up [33].

A telemedicine platform is a means of receiving medical services remotely through various communication technologies [50]. It enables remote communication and data sharing between doctors and patients, thus providing digital medical services for patients after CVR, such as web-based consultation, real-time communication, and disease management [34,37,40,43]. Patients can use portable devices to measure INR at home and transmit the results to the telemedicine platforms. Doctors can then adjust the dosage of anticoagulants and the treatment plan remotely and determine the changes in anticoagulants and conditions of patients after CVR on time [28-30,35]. Moreover, telemedicine platforms can help to supervise the patient’s self-management regime at home for a long time, helping patients correctly understand and implement treatment plans and improving treatment compliance and outcomes [28,29,31,37].

Mobile apps are services that run on smartphones, tablets, or other mobile devices. Some have been developed specifically for AM [36,41,42,46], while others are smartphone-based messaging apps [45,47]. In the research we included, mobile apps mainly mentioned the following four services: (1) Medication assistant: the system automatically generated an oral warfarin regimen based on the patient’s latest INR value and previous warfarin dose, which were reviewed by specialists and sent to patients [42,46]. At the same time, the apps set intelligent reminders every day to urge the patient to take medicines regularly [36,41]. (2) Digital consultation: patients could communicate with doctors on the internet about their condition and anticoagulation treatment [47]. (3) Push health information: apps could send videos or pictures to help patients understand disease-related information [41,42]. (4) Patients’ interactive community: the patients could communicate with other postoperative patients, share their own rehabilitation experiences, and gain support and encouragement [41].

### Application Effects of eHealth Tools in AM

Comprehensive considering the evaluation indexes of anticoagulation effects on patients after CVR can provide better scientific guidance for clinicians and patients. In the literature included, the evaluation of eHealth tools in AM mainly involved three aspects. (1) Clinical outcome: This indicator was reported in 24 papers, including TTR (n=19) [23-28,30,32,33,35-40,42-44,47], rate of achieving target INR (n=5) [27,29,31,42,43], bleeding or thrombotic events (n=18) [23,25,27,28,30,32-42,44,45], and mortality (n=1) [34]. Seven RCTs have demonstrated that using eHealth tools for AM in patients after CVR is a secure and efficacious approach that significantly enhances TTR (*P*<.05) [23,25,26,28,30,32,33]. Computer-based support systems dosing was found to be more effective in improving TTR than medical staff–monitored dosage at the majority of centers (*P*<.001) [24,27,42]. Meanwhile, eHealth tools had a significantly higher number of INR within the target range compared to the conventional administration group (*P*<.05) [27,29]. However, several studies showed no statistical difference (*P>*.05) in the occurrence of bleeding or thrombotic events between the eHealth tools group and the conventional group [23,25,27,30,33]. (2) Economic benefits: Five papers focused on the health economic benefits of eHealth tools, including the frequency of INR tests (n=3) [24,33,43] and cost-effectiveness (n=2) [23,25]. Research showed that the cost of using computerized decision support software was offset by not going to the hospital [23]. However, when considering the costs associated with establishing a nurse-led clinic, the total cost was higher than traditional treatment [25]. In addition, patient self-testing at home could accelerate INR results, but it is not statistically significant in reducing INR test frequency and generating economic benefits [33,34]. (3) Patient satisfaction: The conclusion of 2 studies revealed that most patients were satisfied with the use of eHealth tools (*P*<.001) [23,44], which also improved their quality of life [29,44]. These findings demonstrate the potential of eHealth tools in enhancing AM. However, further studies are warranted to explore the economic benefits of long-term anticoagulant therapy and determine whether it can reduce the incidence of clinical adverse events in patients after CVR.

### Scientometric Analysis

#### Publication Trends

**Figure 2:**
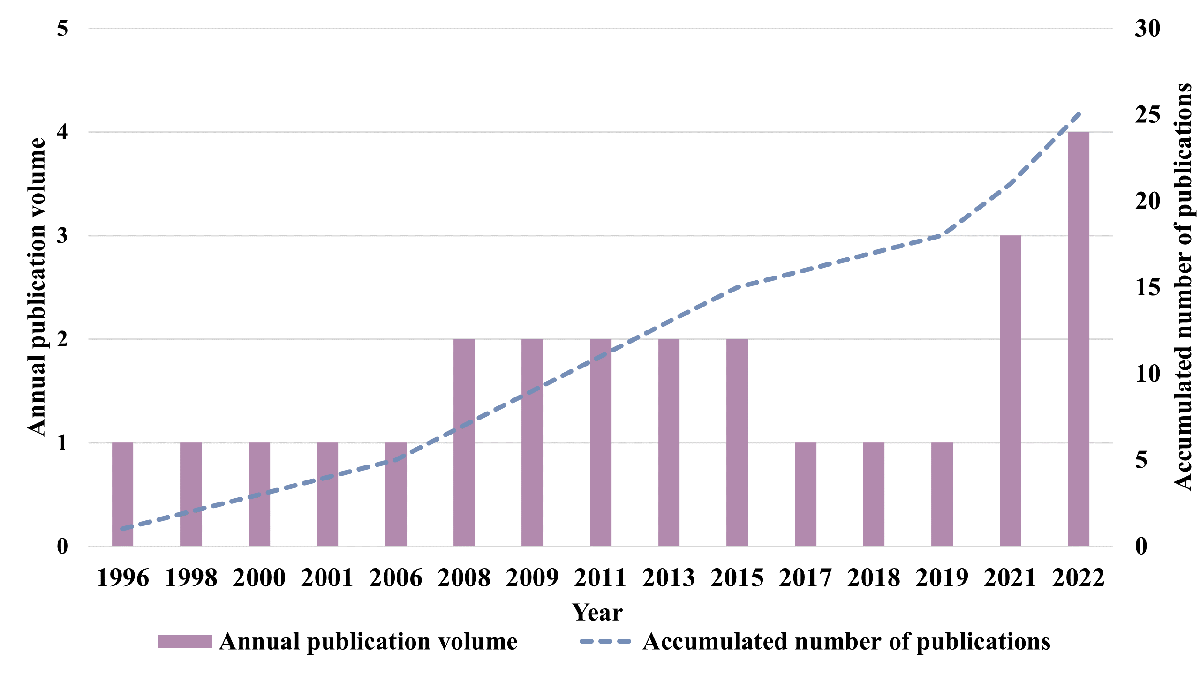
Publication trends of research papers of eHealth tools in anticoagulation management (AM) of patients undergoing cardiac valve replacement (CVR).

The earliest literature on using eHealth tools for AM in patients after CVR was published in 1996. Figure 2 shows the trend of publications, with the number of published papers gradually increasing after 2019. The purple bar above the year label represents the publication volume, and the blue line shows cumulative publication trends. China (n=5) and Italy (n=4) stand out as the 2 countries with the most published papers, the United States and the United Kingdom have each published 3 papers, and other nations have published only 1 or 2 papers. Developed countries had research published around 2000, while transitional countries, such as China and India, only paid attention to this topic around 2019, which may be influenced by cultural differences and medical and economic levels. The majority of the 25 papers in this review were published in thrombus-related journals. However, the application of eHealth tools in the AM of patients after CVR involves not only cardiology and antithrombotic therapy but also the intersection and interdisciplinary cooperation of medical informatics, electronic technology, and health sciences. Some of the papers included in this review were also published in internet medicine journals. Therefore, when seeking the latest progress, readers should not focus only on traditional thrombosis-related journals.

#### Collaborative Analysis of Countries, Research Institutions, and Authors

**Figure 3:**
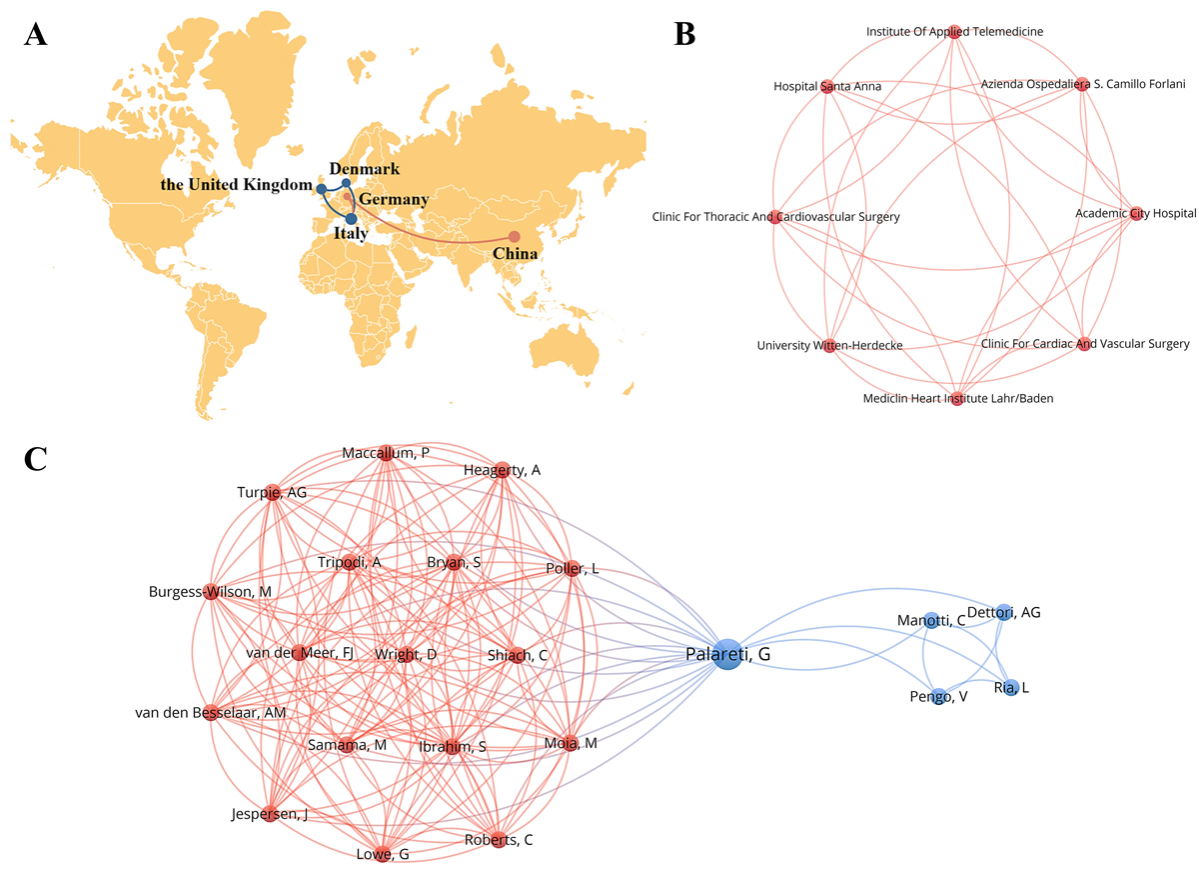
Collaborative network among countries, research institutions, and authors in publications related to the use of eHealth tools for anticoagulation management (AM) in patients after cardiac valve replacement (CVR). (A) Cooperation map among countries. (B) Collaboration network among research institutions. (C) Cooperation network of authors.

The 25 papers selected for this review originated from 12 countries and were associated with 46 institutions. Figure 3A depicts the academic collaboration among 5 of these countries, with China being the only transitional country represented. Each node represents a country, and the connection between nodes represents the cooperative relationship. Figure 3B shows that only 8 of 46 participating institutions show close cooperative relationships, and most of them are from Italy. Each node represents an institution, and the connection between nodes represents the cooperative relationship. It should be noted that there are deficiencies in the exchanges and cooperation between transitional and high-income countries, which poses challenges in enhancing the sharing and complementarity among international academic resources. Furthermore, 89 authors participated in the publication of 25 papers. Each node represents an author, the node size depends on the number of authorial publications, and the different colors of the line represent different cooperation networks (Figure 3C). Two collaborative networks have been formed centered on Palareti G, who is affiliated with the Orsola-Malpighi Hospital in Italy and plays a crucial role in the development of eHealth tools in the AM of patients after CVR.

#### Keyword Co-Occurrence Analysis and Cluster Analysis

**Figure 4:**
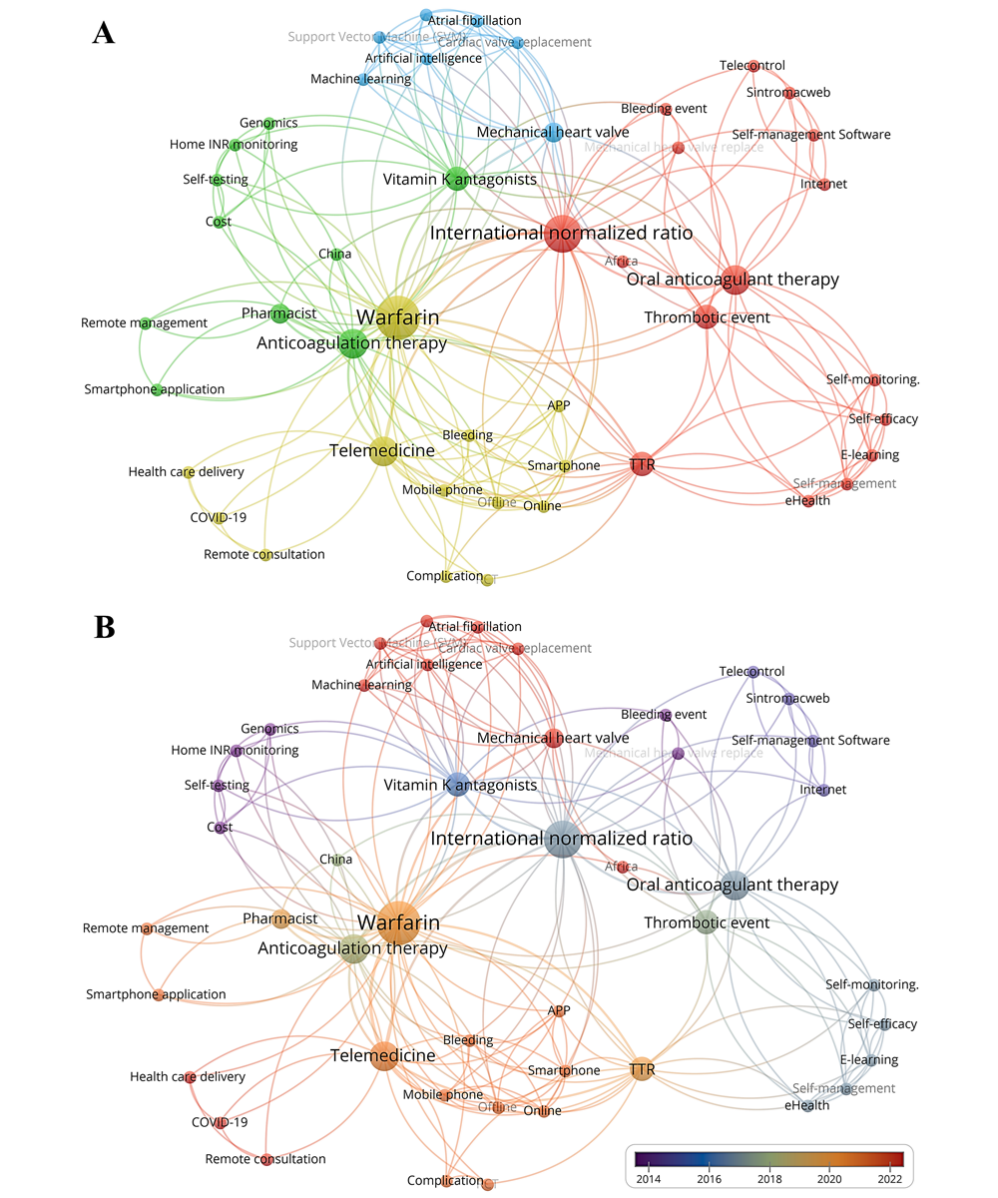
Visualization of keywords of eHealth tools in anticoagulation management (AM) of patients undergoing cardiac valce replacement (CVR). (A) Keywords clustering visualization. (B) Keywords overlay time visualization.

To present the keyword map more clearly, a cluster analysis was conducted for keywords. Figure 4A indicates that current research on the application of eHealth tools in AM after CVR is mainly focused on 4 distinct areas. The node size indicates the frequency of keyword occurrence, and different colors represent different clusters. We found that 16 keywords in the red cluster were closely related to thrombus formation and bleeding, 10 keywords in the green cluster related to the innovation of INR test techniques (home testing, self-testing, etc), keywords in the blue cluster referred to artificial intelligence (AI) and machine learning, and yellow cluster included telemedicine, health care delivery, and remote consultation.

To further explore the hotspots and future directions, we superimposed time on keyword clustering to create keyword overlay visualization (Figure 4B). The larger the node size, the higher the frequency of keywords and the color of nodes corresponded to the average appearance time of keywords. According to high-frequency keyword analysis, anticoagulant drugs for patients undergoing CVR mainly included vitamin K antagonists led by warfarin. Additionally, the research hotspots of eHealth tools in AM have focused on AI, machine learning, and smartphone apps in recent years. Amruthlal et al [46] constructed warfarin dosage prediction models by linear regression, support vector regression, logistic regression, and multilayer perceptron and found that the prediction model based on support vector regression showed the best predictive performance. Support vector regression was installed in a simple user-friendly Android mobile app. The introduction of advanced machine learning algorithms provides more choice and support for AM, which may help to improve the efficiency and accuracy of anticoagulant treatment.

## Discussion

### Summary

This review provides a full evaluation and scientometric analysis of the use of eHealth tools in the AM of patients undergoing CVR surgery. We identified and included 25 studies investigating eHealth tools, such as computer-based support systems, EHRs, telemedicine platforms, and mobile apps. The development of eHealth tools is becoming increasingly important in promoting AM, and the emergence of machine learning and AI has introduced new opportunities for innovation and promotion. Over the past decade, there has been a lack of large-sample and long-term follow-up RCT studies on AM based on eHealth tools, highlighting the need for further research to verify their roles and effects. There remain many areas for improvement to promote the application of eHealth tools in the AM of patients after CVR.

### Key Developments and Benefits Provided by eHealth Tools for AM

The AM of patients after CVR involves a large workload, complex patient information, high technical content, and a long follow-up period. The establishment and application of eHealth tools can improve the efficiency of clinical and management work [36,41,48,49]. The computer-based support system facilitates the standardization of anticoagulant recommendations and avoids differences in the dosage prescribed by medical staff, especially in primary health care institutions lacking anticoagulation therapy experience [51]. However, previously, due to technological limitations, these systems could not closely approximate doctors’ judgment and decision-making skills, and manual verification and intervention were needed to ensure accuracy [24]. With the development of machine learning, the use of deep learning, reinforcement learning, and ensemble learning are increasing gradually in warfarin management after CVR, which can further improve the accuracy of prediction [52,53]. Additionally, doctors can adjust anticoagulant doses of patients more accurately according to their EHR, thus avoiding excessive or insufficient therapy and reducing the occurrence of complications. Although these 2 eHealth tools are highly targeted, easy to operate, and improve work efficiency, their functions are relatively simple and not enough for more comprehensive management of patients undergoing anticoagulant therapy after CVR.

Telemedicine platforms and mobile apps offer a promising solution for expanding access to high-quality medical resources by providing a digital communication channel between clinicians and patients who have undergone CVR [32-36,38,39,41,44]. Through these 2 ways, doctors can provide electronic prescriptions, digital consultations, health education, and self-management support to not only help patients better understand postoperative rehabilitation but also develop their knowledge and skills and improve the compliance, safety, and effectiveness of AM. However, telemedicine platforms depend on high-quality communication devices to be compatible with other medical devices and systems to perform remote processing of medical data and real-time monitoring of patients. In contrast, mobile apps are more portable and can be used anytime and anywhere, with a more intuitive interface and personalized services for anticoagulant therapy [39,44-47]. However, the older population, who comprise the majority of patients undergoing CVR surgery, may have difficulty learning and adapting to mobile apps and data on the internet. This may affect the accuracy of communication and information transfer between doctors and patients and limit postoperative follow-up. Additionally, practical limitations, such as network instability, data security, low degree of automation, and the need for medical institution support, could affect the effectiveness of telemedicine platforms and mobile apps.

### Research on Hotspots of eHealth Tools Based on Scientometric Analysis

Research on the use of eHealth tools for AM after CVR surgery is lacking on a global scale. Developed nations have conducted more studies in this area, which may be attributed to their advanced economic status, superior medical quality, technical proficiency, and greater patient acceptance [54,55]. To promote the development of eHealth tools, it is suggested that countries provide a platform for exchanging and sharing the latest research results and invite scholars from different fields to participate in medical exchange programs, visiting scholar programs, medical conferences, and seminars. Moreover, universities can encourage students to effectively establish a cooperative culture, organize interdisciplinary research groups, conduct interdisciplinary training and education, and cultivate medical talents with an interdisciplinary background and international vision.

Based on the results of keyword clustering and overlay visualization, AI and machine learning have been adopted as research methods for developing eHealth tools and become a hotspot in recent years. These methods have provided valuable insights for future research. Genetic algorithms, backpropagation neural networks, and adapted neural-fuzzy inference system models have the potential to establish more accurate and stable prediction models of warfarin individual maintenance dose for patients after CVR [52,56]. Medical professionals can use biometric technology to visually identify the patient, the medication, and the confirmed ingestion, thereby reducing the risk of noncompliance in patients' anticoagulant therapy [57]. To promote the wide application and sustainable development of eHealth tools, it is necessary to research intelligent auxiliary tools, combining sophisticated machine learning techniques and AI models to enhance the precision and dependability of prediction and innovate anticoagulant therapy tools.

### Future Directions for eHealth Tools in AM of Patients After CVR

In 25 studies included, although the main outcome indicators of eHealth tools in AM covered most of the clinical indicators, they could not fully reflect the overall situation of patients from the perspectives of economy, society, psychology, and user experience. Therefore, it is suggested that improvements can be made in the following aspects: (1) Patient interaction indicators: Researchers can monitor the frequency of patients logging in or using eHealth tools in the background, learn about their experience and needs, and then adjust the tool design and functions accordingly. For example, a user interface with voice support, large font, and novice guidance can help patients overcome the potential obstacles in using eHealth tools [58]. (2) Indicators of acceptability: Researchers should evaluate the acceptance of patients and health care providers using patient-physician satisfaction, use rate, and patient-medication compliance. (3) Anticoagulant dosage index: This can be used to evaluate the accuracy and consistency of manual administration and eHealth tool–assisted administration to ensure the correctness of AM decisions. (4) Sustainability indicators: Patients can evaluate the user experience of eHealth tools from engagement, function, esthetics, and information quality to ensure long-term use and promotion [59].

In addition to the aforementioned optimization metrics, several other important factors should be considered to facilitate eHealth tools. First, it is critical to ensure that the design of eHealth tools is compatible with other medical devices and systems that the patients may use (eg, blood clotting machines and electrocardiographs). To minimize errors and improve data accuracy, these tools should connect seamlessly, allowing data to be shared and integrated across different platforms. Lubitz et al [60] used compatible, wearable devices and Android or iOS smartphones–assisted electrocardiogram patch monitoring, which effectively identified undiagnosed atrial fibrillation at an early stage. Second, providing effective training and support to older or less educated patients is critical for increasing the acceptance and use of eHealth tools. Previous studies have shown that tailored coaching and education programs can help patients operate these tools effectively [61]. Third, the use of advanced machine learning algorithms and AI models helps further improve the accuracy and reliability of eHealth tools. Zeng et al [62] used various machine learning to construct a dynamic anticoagulant treatment scheme for hospitalized patients after CVR surgery. The results showed that the performance of reinforcement learning was significantly better than the other algorithms, and the quality of anticoagulation was significantly optimized [62]. Finally, implementing effective evaluation mechanisms for patients lost to follow-up is critical to ensure they receive appropriate anticoagulant treatment over the long term. Porter et al [63] conducted a 2-year follow-up of patients receiving oral anticoagulants and found that 12-week INR test intervals were feasible, which saved patient’s time and improved their compliance [63]. By addressing these factors and optimizing the application of eHealth tools in the AM of patients after CVR, it is possible to significantly improve the overall therapeutic effect and reduce the risk of complications.

### Limitations

This review has some limitations. First, we did not assess the quality of the included studies because the study was to provide a comprehensive overview of the existing research landscape related to the application of eHealth tools in AM for patients after CVR. However, we believe that evaluating the quality of the literature will increase the significance of the study. Second, we did not include studies published in the form of other sources, such as qualitative studies, because we were unable to identify eligible studies. Third, concluding trends based on 25 papers are limited, and the conclusions of scientometric analysis should be interpreted with caution. The scientometric analysis in our research is influenced by several limitations, such as the omission of qualitative data, language barriers, and the potential for sample bias. To address the constraints associated with scientometric analysis, researchers can broaden their data sources, integrate qualitative data, and encompass papers published in various languages.

### Conclusions

The application of AM based on eHealth tools is expected to truly reflect the social and economic benefits of digital intelligence, thus benefiting patients undergoing CVR. This study provides an overview of the scope, benefits, and future development of eHealth tools in AM for researchers, health care professionals, and post-CVR patients. However, the economic benefits and long-term impact of adverse events need further explored. It is suggested that future eHealth tools in AM should concentrate on enhancing patient contact, acceptance, cost-effectiveness, and sustainability while combining sophisticated algorithms to enhance the precision and dependability of eHealth tools.

## Appendix

Multimedia Appendix 1 Preferred Reporting Items for Systematic Reviews and Meta-Analyses extension for Scoping Reviews.

Multimedia Appendix 2 Search strategy per database.

Multimedia Appendix 3 Summary of the study characteristics.

## Abbreviations

AI: artificial intelligence
AM: anticoagulation management
CVR: cardiac valve replacement
EHR: electronic health record
INR: international normalized ratio
RCT: randomized controlled trial
PRISMA-ScR: Preferred Reporting Items for Systematic Reviews and Meta-Analyses extension for Scoping Review
TTR: time in therapy range

## Data Availability

The data sets used and analyzed during this study are available from the corresponding author on reasonable request.
