# Supplementary material for: Application of eHealth Tools in Anticoagulation Management After Cardiac Valve Replacement: Scoping Review Coupled With Bibliometric Analysis
Source: JMIR Mhealth Uhealth. 2024 Jan 5;12:e48716. doi: 10.2196/48716 (PMC10799280; doi:10.2196/48716)
Supplement: Multimedia Appendix 2 [file mhealth_v12i1e48716_app2.docx]

173 Pubmed: 1950 to March 1, 2023

#1: (anticoagulants[Title/Abstract]) OR (warfarin[Title/Abstract]) OR (warfarin therapy[Title/Abstract]) OR (decoagulant[Title/Abstract]) OR (anticoagulation agents[Title/Abstract]) OR (anticoagulant drugs[Title/Abstract]) OR (indirect thrombin inhibitors[Title/Abstract]) OR (oral anticoagulants[Title/Abstract]) OR (direct oral anticoagulants[Title/Abstract]) OR (antithrombotics[Title/Abstract]) OR (anticaking agent[Title/Abstract]) OR (anticoagulation[Title/Abstract]) OR (anticoagulation therapy[Title/Abstract]) OR (anticoagulation treatment[Title/Abstract]) OR (anticoagulant management[Title/Abstract]) OR (anticoagulant rehabilitation [Title/Abstract]) OR (anticoagulation services[Title/Abstract]) OR (international normalized ratio[Title/Abstract]) OR (postoperative management [Title/Abstract]) OR (apo-warfarin[Title/Abstract]) OR (aldocumar[Title/Abstract]) OR (gen-warfarin[Title/Abstract]) OR (coumadin[Title/Abstract]) OR (warfarin potassium[Title/Abstract]) OR (warfarin sodium[Title/Abstract]) OR (coumadine[Title/Abstract]) OR (direct thrombin inhibitors[Title/Abstract]) OR (argatroban[Title/Abstract]) OR (dabigatran[Title/Abstract]) OR (rivaroxaban[Title/Abstract]) OR (apixaban[Title/Abstract]) OR (edoxaban[Title/Abstract]) OR (platelet aggregation inhibitors[Title/Abstract]) OR (platelet inhibitors[Title/Abstract]) OR (antiplatelet agents[Title/Abstract]) OR (thrombin inhibitors[Title/Abstract]) OR (vitamin K antagonists[Title/Abstract]) OR (INR monitoring) OR (home INR testing[Title/Abstract])

#2: (Telemedicine[mesh terms]) or (mobile health) or (mHealth) or (telehealth) or (eHealth) or (teleconsultation) or (mobile technology) or (telecare) or (internet) or (digital health) or (smartphone) or (APP) or (mobile application) or (portable electronic apps) or (digital) or (smart) or (intelligent) or (mobile) or (telehealth) or (artificial intelligence) or (computer systems) or (electronic health records) or (portable electronic applications) or (remote patient monitoring) or (telemedicine platforms) or (healthcare software) or (healthcare devices) or (healthcare systems) or (wearable technology) or (remote monitoring) or (healthcare analytics) or (patient portals) or (virtual reality) or (augmented reality) or (health tracking devices) or (health apps) or (health gadgets) or (digital therapeutics) or (health coaching apps) or (personal health records) or (telemedicine robots) or (smart home health devices) or (connected health devices) or (health chatbots) or (voice assistants for healthcare) or (digital health platforms) or (health data analytics) or (smart medical devices)

#3: (Heart valve prosthesis implantation[Mesh]) or (aortic valve replacement) or (heart valve surgery) or (cardiac valve replacement) or (mitral valve replacement) or (mitral valvuloplasty) or (tricuspid valve replacement) or (pulmonary valve replacement) or (valvotomy) or (percutaneous valvular intervention) or (prosthetic heart valve insertion) or (artificial valve replacement) or (aortic valve stenosis) or (transcatheter aortic valve replacement) or (valvular heart disease) or (cardiovascular surgical procedures) or (surgical procedures, operative) or (prosthesis implantation) or (cardiac surgical procedures) or (thoracic surgical procedures) or (surgery, cardiac) or (surgery, heart) or (heart surgery) or (cardiac surgery) or (procedure, cardiac surgical) or (procedures, cardiac surgical) or (surgical procedure, cardiac) or (surgical procedures, cardiac) or (surgical procedures, heart) or (cardiac surgical procedure) or (heart surgical procedures) or (procedure, heart surgical) or (procedures, heart surgical) or (surgical procedure, heart) or (heart surgical procedure) or (cardiac valve annuloplasty)

#4: #1 and #2

#5: #4 and #3

Filters: Clinical Trial, Randomized Controlled Trial, English

110 WOS: 1953 to March 1, 2023

((((TI=(anticoagulants OR warfarin OR warfarin therapy OR decoagulant OR anticoagulation agents OR anticoagulant drugs OR indirect thrombin inhibitors OR oral anticoagulants OR antithrombotics OR anticaking agent OR anticoagulation OR anticoagulation therapy OR anticoagulation treatment OR anticoagulant management OR anticoagulation services OR international normalized ratio OR direct oral anticoagulants OR apo-warfarin OR aldocumar OR gen-warfarin OR warfant OR coumadin OR warfarin potassium OR warfarin sodium OR coumadine OR warfarin potassium OR warfarin sodium OR coumadine OR direct thrombin inhibitors OR argatroban OR dabigatran OR rivaroxaban OR apixaban OR edoxaban OR platelet aggregation inhibitors OR platelet inhibitors OR antiplatelet agents OR thrombin inhibitors OR vitamin K antagonists OR INR monitoring OR home INR testing)) AND TI=(*prosthesis implantation OR *valve replacement OR heart valve* OR *valvuloplasty OR *valvotomy OR percutaneous valvular intervention OR aortic valve stenosis OR valvular heart* OR cardiovascular surgical procedures OR cardiac surgical procedures OR prosthesis implantation OR thoracic surgical procedures OR valve replace* OR valve implant* OR valvuloplasty* OR valve prosthesis OR aortic* OR mitral* OR tricuspid*)) AND TS=(telemedicine* OR mobile* OR smart* OR mHealth OR eHealth OR tele* OR internet OR digital* OR APP OR application OR portable electronic* OR healthcare software OR intelligent* OR remote monitoring OR artificial intelligence OR computer* OR electronic health* OR intelligent* OR healthcare systems))) AND ((LA==("ENGLISH") NOT (SILOID==("PPRN") OR DT==("ABSTRACT" OR "MEETING" OR "CASE REPORT" OR "EDITORIAL MATERIAL" OR "LETTER" OR "PATENT" OR "NEWS"))))

68 EMBASE: 1950 to March 1, 2023

('anticoagulants':ti,ab,kw OR 'warfarin':ti,ab,kw OR 'warfarin therapy':ti,ab,kw OR 'decoagulant':ti,ab,kw OR 'anticoagulation agents':ti,ab,kw OR 'anticoagulant drugs':ti,ab,kw OR 'indirect thrombin inhibitors':ti,ab,kw OR 'oral anticoagulants':ti,ab,kw OR 'antithrombotics':ti,ab,kw OR 'anticaking agent':ti,ab,kw OR 'anticoagulation':ti,ab,kw OR 'anticoagulation therapy':ti,ab,kw OR 'anticoagulation treatment':ti,ab,kw OR 'anticoagulant management':ti,ab,kw OR 'anticoagulation services':ti,ab,kw OR 'direct oral anticoagulants':ti,ab,kw OR 'apo-warfarin':ti,ab,kw OR 'coumadine':ti,ab,kw OR 'warfarin potassium':ti,ab,kw OR 'warfarin sodium':ti,ab,kw OR 'direct thrombin inhibitors':ti,ab,kw OR 'argatroban':ti,ab,kw OR 'dabigatran':ti,ab,kw OR 'rivaroxaban':ti,ab,kw OR 'apixaban':ti,ab,kw OR 'edoxaban':ti,ab,kw OR 'platelet aggregation inhibitors':ti,ab,kw OR 'platelet inhibitors':ti,ab,kw OR 'antiplatelet agents':ti,ab,kw OR 'thrombin inhibitors':ti,ab,kw OR 'vitamin k antagonists':ti,ab,kw OR 'inr monitoring':ti,ab,kw OR 'home inr testing':ti,ab,kw) AND ('heart valve prosthesis implantation':ti,ab,kw OR 'aortic valve replacement':ti,ab,kw OR 'heart valve surgery':ti,ab,kw OR 'cardiac valve replacement':ti,ab,kw OR 'mitral valve replacement':ti,ab,kw OR 'mitral valvuloplasty':ti,ab,kw OR 'tricuspid valve replacement':ti,ab,kw OR 'pulmonary valve replacement':ti,ab,kw OR valvotomy:ti,ab,kw OR 'percutaneous valvular intervention':ti,ab,kw OR 'prosthetic heart valve insertion':ti,ab,kw OR 'artificial valve replacement':ti,ab,kw OR 'aortic valve stenosis':ti,ab,kw OR 'transcatheter aortic valve replacement':ti,ab,kw OR 'valvular heart disease':ti,ab,kw OR 'cardiovascular surgical procedures':ti,ab,kw OR 'surgical procedures, operative':ti,ab,kw OR 'prosthesis implantation':ti,ab,kw OR 'cardiac surgical procedures':ti,ab,kw OR 'thoracic surgical procedures':ti,ab,kw OR 'surgery, cardiac':ti,ab,kw OR 'surgery, heart':ti,ab,kw OR 'heart surgery':ti,ab,kw OR 'cardiac surgery':ti,ab,kw OR 'procedure, cardiac surgical':ti,ab,kw OR 'procedures, cardiac surgical':ti,ab,kw OR 'surgical procedure, cardiac':ti,ab,kw OR 'surgical procedures, cardiac':ti,ab,kw OR 'surgical procedures, heart':ti,ab,kw OR 'cardiac surgical procedure':ti,ab,kw OR 'heart surgical procedures':ti,ab,kw OR 'procedure, heart surgical':ti,ab,kw OR 'procedures, heart surgical':ti,ab,kw OR 'surgical procedure, heart':ti,ab,kw OR 'heart surgical procedure':ti,ab,kw OR 'cardiac valve annuloplasty':ti,ab,kw) AND ('telemedicine':ti,ab,kw OR 'mobile':ti,ab,kw OR 'smart':ti,ab,kw OR 'mhealth':ti,ab,kw OR 'ehealth':ti,ab,kw OR 'tele':ti,ab,kw OR 'internet':ti,ab,kw OR 'digital':ti,ab,kw OR 'app':ti,ab,kw OR 'portable electronic':ti,ab,kw OR 'intelligent':ti,ab,kw OR 'artificial intelligence':ti,ab,kw OR 'computer':ti,ab,kw OR 'electronic health*':ti,ab,kw OR 'remote patient monitoring':ti,ab,kw OR 'telemedicine platforms':ti,ab,kw OR 'healthcare software':ti,ab,kw OR 'healthcare devices':ti,ab,kw OR 'healthcare systems':ti,ab,kw OR 'wearable technology':ti,ab,kw OR 'remote monitoring':ti,ab,kw OR 'healthcare analytics':ti,ab,kw OR 'patient portals':ti,ab,kw OR 'virtual reality':ti,ab,kw OR 'augmented reality':ti,ab,kw OR 'health tracking devices':ti,ab,kw OR 'health apps':ti,ab,kw OR 'health gadgets':ti,ab,kw OR 'digital therapeutics':ti,ab,kw OR 'health coaching apps':ti,ab,kw OR 'personal health records':ti,ab,kw OR 'telemedicine robots':ti,ab,kw OR 'smart home health devices':ti,ab,kw OR 'connected health devices':ti,ab,kw OR 'health chatbots':ti,ab,kw OR 'voice assistants for healthcare':ti,ab,kw OR 'digital health platforms':ti,ab,kw OR 'health data analytics':ti,ab,kw OR 'smart medical devices':ti,ab,kw) AND [english]/lim AND 'article'/it

73 CLNLICAL: 1984 to March 1, 2023

"( (Anticoagulants) or (warfarin) or (warfarin therapy) or (decoagulant) or (anticoagulation agents) or (anticoagulant drugs) or (indirect thrombin inhibitors) or (oral anticoagulants) or (direct oral anticoagulants) or (antithrombotics) or (anticaking agent) or (anticoagulation) or (anticoagulation therapy) or (anticoagulation treatment) or (anticoagulant management) or (anticoagulation services) or (international normalized ratio) or (rehabilitation) or (apo-warfarin) or (gen-warfarin) or (coumadin) or (warfarin potassium) or (warfarin sodium) or (coumadine) or (warfarin potassium) OR (warfarin sodium ) OR (coumadine ) OR (direct thrombin inhibitors ) OR (argatroban ) OR (dabigatran ) OR (rivaroxaban ) OR (apixaban ) OR (edoxaban ) OR (platelet aggregation inhibitors ) OR (platelet inhibitors ) OR (antiplatelet agents ) OR (thrombin inhibitors ) OR (vitamin K antagonists ) OR (INR monitoring) OR (home INR testing ) ) AND ( (Telemedicine) or (mobile health) or (mHealth) or (telehealth) or (eHealth) or (teleconsultation) or (mobile technology) or (telecare) or (internet) or (digital health) or (smartphone) or (APP) or (mobile application) or (portable electronic apps) or (digital) or (smart) or (intelligent) or (mobile) or (telehealth) or (artificial intelligence) or (computer systems) or (electronic health records) or (portable electronic applications) or (remote patient monitoring) or (telemedicine platforms) or (healthcare software) or (healthcare devices) or (healthcare systems) or (wearable technology) or (remote monitoring) or (healthcare analytics) or (patient portals) or (virtual reality) or (augmented reality) or (health tracking devices) or (health apps) or (health gadgets) or (digital therapeutics) or (health coaching apps) or (personal health records) or (telemedicine robots) or (smart home health devices) or (connected health devices) or (health chatbots) or (voice assistants for healthcare) or (digital health platforms) or (health data analytics) or (smart medical devices) ) AND ( (Heart valve prosthesis implantation) or (aortic valve replacement) or (heart valve surgery) or (cardiac valve replacement) or (mitral valve replacement) or (mitral valvuloplasty) or (tricuspid valve replacement) or (pulmonary valve replacement) or (valvotomy) or (percutaneous valvular intervention) or (prosthetic heart valve insertion) or (artificial valve replacement) or (aortic valve stenosis) or (transcatheter aortic valve replacement) or (valvular heart disease) or (cardiovascular surgical procedures) or (surgical procedures, operative) or (prosthesis implantation) or (cardiac surgical procedures) or (thoracic surgical procedures) or (surgical procedures, operative) or (prosthesis implantation) or (cardiac surgical procedures) or (thoracic surgical procedures) or (surgery, cardiac) or (surgery, heart) or (heart surgery) or (cardiac surgery) or (procedure, cardiac surgical) or (procedures, cardiac surgical) or (surgical procedure, cardiac) or (surgical procedures, cardiac) or (surgical procedures, heart) or (cardiac surgical procedure) or (heart surgical procedures) or (procedure, heart surgical) or (procedures, heart surgical) or (surgical procedure, heart) or (heart surgical procedure) or (cardiac valve annuloplasty) )

92 MEDLINE: 1950 to March 1, 2023

((((TI=(anticoagulants OR warfarin OR warfarin therapy OR decoagulant OR anticoagulation agents OR anticoagulant drugs OR indirect thrombin inhibitors OR oral anticoagulants OR antithrombotics OR anticaking agent OR anticoagulation OR anticoagulation therapy OR anticoagulation treatment OR anticoagulant management OR anticoagulation services OR international normalized ratio OR direct oral anticoagulants OR apo-warfarin OR aldocumar OR gen-warfarin OR warfant OR coumadin OR warfarin potassium OR warfarin sodium OR coumadine OR warfarin potassium OR warfarin sodium OR coumadine OR direct thrombin inhibitors OR argatroban OR dabigatran OR rivaroxaban OR apixaban OR edoxaban OR platelet aggregation inhibitors OR platelet inhibitors OR antiplatelet agents OR thrombin inhibitors OR vitamin K antagonists OR INR monitoring OR home INR testing)) AND TI=(*prosthesis implantation OR *valve replacement OR heart valve* OR *valvuloplasty OR *valvotomy OR percutaneous valvular intervention OR aortic valve stenosis OR valvular heart* OR cardiovascular surgical procedures OR cardiac surgical procedures OR prosthesis implantation OR thoracic surgical procedures OR valve replace* OR valve implant* OR valvuloplasty* OR valve prosthesis OR aortic* OR mitral* OR tricuspid*)) AND TS=(telemedicine* OR mobile* OR smart* OR mHealth OR eHealth OR tele* OR internet OR digital* OR APP OR application OR portable electronic* OR healthcare software OR intelligent* OR remote monitoring OR artificial intelligence OR computer* OR electronic health* OR intelligent* OR healthcare systems))) AND ((LA==("ENGLISH") NOT (SILOID==("PPRN") OR DT==("ABSTRACT" OR "MEETING" OR "CASE REPORT" OR "EDITORIAL MATERIAL" OR "LETTER" OR "PATENT" OR "NEWS"))))
