# Supplementary material for: Application of eHealth Tools in Anticoagulation Management After Cardiac Valve Replacement: Scoping Review Coupled With Bibliometric Analysis
Source: JMIR Mhealth Uhealth. 2024 Jan 5;12:e48716. doi: 10.2196/48716 (PMC10799280; doi:10.2196/48716)
Supplement: Multimedia Appendix 3 [file mhealth_v12i1e48716_app3.docx]

**Multimedia Appendix 3**

Table S1. Summary of the study characteristics (N=25)

| Author, Year, Country | Study design | Study objective | Participants characteristics | Intervention/  follow-up times | Intervention method | Main Outcome and Results | Conclusion |
| --- | --- | --- | --- | --- | --- | --- | --- |
| Fitzmaurice et al., 1996; United Kingdom | RCT^a^ | To evaluate the effectiveness of computerized decision support in OA^b^ therapy in primary care settings. | Sample: (N=49)  Age: Not reported  Gender: Not reported  Valve type: mechanical valve  Loss to follow-up: - | 12 months | 1. Intervention group: received computer support.  2. Control group: standard care and adjustment by the treating physician. | 1. TTR^c^_:_ There were significant improvements in INR^d^ control from 23% to 86% (P<0.001).  2. Adverse events: No significant differences. 3. Cost-Effectiveness: The costs of the DSS^e^ were offset by savings from patients not attending the hospital clinic. 4. Patient satisfaction: The practice clinic was high. | Computerized DSS enables the safe and effective transfer of AM from hospital to primary care and may result in improved patient outcomes in terms of the level of control, frequency of review, and general acceptability. |
| Ageno et al.,  1998; Canada | RCT | To assess the accuracy and clinical utility of a computer-based dosage program as compared with manual dosing in monitoring outpatients on long-term OA therapy after mechanical heart valve replacement. | Sample: (N=101)  Age: Not reported  Gender: Not reported  Valve type: mechanical valve  Loss to follow-up: - | 10 months | 1. Intervention group: received computer dosing.  2. Control group: standard care with manual dosing by clinic staff. | 1. TTR: No significant differences.  2. Dose changes: patients were 410 in the manual group and 221 in the computer group. 3. INR tests: 16.9 in the manual group and 14.1 in the computer group. | The effectiveness and clinical application value of computer programs in improving anticoagulation control were proved. Human intervention is needed to check the results of the computerized system. |
| Fitzmaurice et al.,  2000; United Kingdom | RCT | To assess if OA care in primary care, through a nurse-led clinic with near-patient testing and computerized decision support software, is comparable to routine hospital management. | Sample: (N=367)  Age: Not reported  Gender: Not reported  Valve type: mechanical valve  Loss to follow-up:55 | 12 months | 1. Intervention group: received the AM^f^ of computerized decision support systems and near-patient testing.  2. Control group: routine AM in the hospital. | 1. TTR: Significant improvement in the intervention group (P=0.008).  2. Adverse events: No significant differences.  3. Cost-Effectiveness: The intervention group was about £100 more per patient per year than the control group. | Nurse-led anticoagulation clinics can be implemented in novice primary care settings using computerized decision support software and near-patient testing. |
| Manotti et al.,  2001; Italy | RCT | To test whether a computer-based decision support system to initiate and maintain OA treatment can improve the laboratory quality of therapy. | Sample: (N=1358)  Mean age: 66.8 years  Male: 691 Female:667  Valve type: mechanical valve and bioprosthetic valves  Loss to follow-up: - | 3 months | 1. Intervention group: computer-aided dosing  2. Control group: expert physicians dosing | 1. TTR: Patients in the computer-aided dosing group achieved a stable state significantly faster (P<0.001) and they spent more time within the TTR during maintenance than controls (P<0.001). | Computer decision-aided support improves the laboratory quality of anticoagulant treatment in maintenance and unstable phases, significantly reducing scheduled lab controls. |
| Testa et al.,  2006; Italy | Pilot Study | To establish a telemedicine system to decentralize AM in local medical centers. | Sample: (N=447)  Age: Not reported  Male: 225 Female:179  Valve type: unspecified  Loss to follow-up: 43 | 12 months | TaoNet collected and analyzed patient clinical data, connected with the peripheral health units and AC^g^. | 1. TTR  A significant increase in TTR (AC = 62%, peripheral health units = 70%).  2. Adverse events  No significant differences. | The telemedicine system enables direct communication between the surrounding health unit and the AC, and improves patient satisfaction, quality of life, and reduces the number of laboratory tests for patients. |
| O'Shea et al.,  2008; USA | Cohort study | To determine whether an interactive, internet-based system enabling supervised, patient self-management of OA therapy provided management comparable to an established anticoagulation clinic. | Sample: (N=60)  Median age: 54.1 years  Male: 28 Female:30  Valve type: unspecified  Loss to follow-up: 2 | 10 months | Patients used devices to obtain their INR values. They then go to the website to input their health information, and doctors review patient data and management plans via the Internet. | 1. TTR:  The TTR increased from 63% to 74.4% during internet-supervised patient self-management  2. Adverse events  No significant differences. | The demonstration of an internet-based expert system can remotely and effectively supervise patients' self-management and improve TTR. |
| Poller et al.,  2008; United Kingdom | RCT | To compare the safety and effectiveness of computer-assisted dosage with dosage by experienced medical staff at the same centers. | Sample: (N=13219)  Age: Mainly over 50 years  Male: 6990 Female:5893 Not informed: 169  Valve type: mechanical valve  Loss to follow-up: 167 | 54 months | 1. Intervention group: computer-aided dosing.  2. Control group: manual dosage. | 1. TIR^h^  TIR was significantly improved by computer assistance as compared with medical staff dosage (P < 0.001).  2. Adverse events  No significant differences. | The safety and effectiveness of computer-assisted dosage have been demonstrated using two different marketed programs in comparison with experienced medical staff dosage. |
| Soliman et al.,  2009; Netherlands | RCT | To explore the impact of anticoagulant site-based self-management on anticoagulant therapy control and quality of life compared to traditional Dutch thrombosis services. | Sample: (N=62)  Mean age: 56 years  Gender: Not reported  Valve type: mechanical valve Loss to follow-up: 4 | 29 months | 1. Intervention group: self-test INR and communicate with doctors through the website.  2. Control group: controlled by the local thrombosis service. | 1. TIR  The number of TIR was significantly higher in the self-management group (P = 0.01).  2. Quality of life scores  The significant differences in the pain, vitality, and role emotional (P =0.01). | Further studies are needed to describe whether the self-management program will reduce the risk of bleeding and/or thromboembolism. |
| Ryan et al.,  2009; Ireland | RCT | To test the hypothesis that supervised patient self-testing using an internet-based expert system could provide superior anticoagulation control to that provided by routine AM. | Sample: (N=162)  Mean age: 58.7 ± 14.3years  Male: 80 Female:52  Valve type: mechanical valve Loss to follow-up: 30 | 12 months | 1. Intervention group: patient self-testing and received instant feedback through software systems.  2. Control group: pharmacist or physician dosage. | 1. TTR  TTR was significantly higher during patient self-testing management (median TTR 74% vs 58.6%, P < 0.001).  2. Adverse events  No significant differences. | The use of an internet-based, direct-to-patient expert system for the management of patient self-testing improves the control of oral anticoagulation therapy. |
| Christensen  et al., 2011; Denmark. | RCT | To investigate if the weekly measurement and dosing of INR at home using the online Internet-based system was superior to conventional treatment. | Sample: (N=140)  Age: Mainly 60 years  Male: 92 Female: 31  Valve type: mechanical valve  Loss to follow-up: 17 | Group A: 514 months  Group B: 411 months  Control group:  473 months | Group A: once weekly measurement and report INR online.  Group B: twice weekly measurement and report INR online  Control group: conventional treatment with INR measurement in the lab. | 1. TTR  Groups A and B were significantly better than the conventional group C, with a difference of TTR of 7% points (P < 0.05). No difference was seen between A and B.  2. Adverse events  No significant differences. | Telemedicine and home-based INR measurements to manage patients' therapy are safe and may result in better quality. |
| Cafolla et al.,  2011; Italy | Cohort study | To verify the increased quality of therapy by time spent in INR target and efficiency and safety of Zeus algorithm. | Sample: (N=1876)  Age: Mainly 40 to 80 years  Male: 974 Female:902  Valve type: mechanical valve  Loss to follow-up: 92 | 24 months | 1. Intervention group: Computer-aided medication based on the new Zeus algorithm.  2. Control group: manual dosage. | 1. TTR:  TTR was significantly higher during the algorithm dosing in comparison with manual management (62.3% vs 50.3%, (P<0.001).  2. Adverse events  Zeus group was significantly lower than the manual dosage (P<0.05). | The “Zeus” dosing algorithm was effective in improving and standardizing the AM quality in maintenance phase patients. |
| Thompson et al.,  2013; USA | RCT | To compare the in-hospital initiation of international normalized ratio self-testing with usual care in mechanical heart valve recipients. | Sample: (N=200)  Median age: 54 years  Male: 127 Female:73  Valve type: mechanical valve  Loss to follow-up: 38 | 3 months | 1. Intervention group: Self-test and INR input into accountability Act–compliant automated computer system.  2. Control group: manual dosage. | 1. TTR:  TTR was significantly higher in the self-testing group 52% vs the usual-care group 45% (P=0.05).  2. Adverse events  No significant differences.  3. The frequency of INR tests  No significant differences. | Self-testing patients reported being more comfortable with how their INR was monitored, and the difference in time needed to obtain an INR value using self-testing versus usual care was striking. |
| Bussey et al.,  2013; USA | Pilot Study | To evaluate the effect of a system combining frequent INR self-testing with online remote monitoring and management and low-dose vitamin K supplementation on INR control. | Sample: (N=55)  Age (median): 63 years  Male: 33 Female:22  Valve type: mechanical valve  Loss to follow-up: 8 | 12 months | 1. Intervention group: self-testing with online remote monitoring and management.  2. Control group: clinician management. | 1. TTR:  TTR improved from 56% to 81% after the intervention (P<0.001).  2. Extreme INR  Time spent at INR lower than 1.5 or higher than 5 was reduced from 3.1% to 0.4% (P=0.01).  3. The frequency of INR tests  No significant differences. | The 25% increase in TTR with online remote monitoring and management was greater than other previously reported measures to improve TTR and required minimal clinician time. |
| Koertke et al.,  2015; Germany | RCT | To investigate the efficacy and safety of patients performing INR self-control under telemedicine guidance. | Sample: (N=1571)  Age: 18 to 81 years  Male: 1122 Female:449  Valve type: mechanical valve  Loss to follow-up: 363 | 24 months | 1. Very low-dose group: INR control once a week (VLO)  2. Very low-dose group: INR control twice a week (VLT)  3. Low-dose group (LOW): Patient self-control of INR. | 1. Overall mortality  The 2-year mortality was 1.685 (P=0.421) for the VLO group and 4.7 (P=0.004) for the VLT group.  2. Adverse events  Freedom from major bleeding in the LOW, VLO, and VLT group was 96.3%, 98.6%, and 99.1% (P=0.008), thrombosis was 99%, 99.8%, and 98.9%, respectively (P =0.258). | Telemedicine-guided very low-dose INR self-control is comparable with low-dose INR in thrombotic risk and is superior in bleeding risk. Weekly testing is sufficient. |
| Ferrando et al.,  2015; Spain | Cohort study | To assess the effectiveness and safety of OA therapy management in patients using the Sintromac Web tele-control system about control in patients using the conventional system. | Sample: (N=175)  Mean age: 64 years  Male: 88 Female:87  Valve type: mechanical valve Loss to follow-up: 2 | 6-8months | 1. Intervention group: tele-control with Sintromac Web.  2. Control group: conventional control. | 1. TTR  TTR was significantly higher in the tele-control group than in the control group (62% vs. 54%, P=0.01).  2. Adverse events  Thrombotic and/or bleeding events occurred in 11 patients of the control control group, and 3 patients of the tele-control group (P=0.03). | In clinical practice, OA management with the Sintromac Web is effective and safe for those patients who are eligible for OA tele-control. |
| Talboom et al., 2017; Netherlands | Cohort study | To analyze the effect on anticoagulation control of an intervention consisting of an education program in combination with the use of an online self-management portal. | Sample: (N=247)  Mean age: 66.9 years  Male: 133 Female:114  Valve type: mechanical valve Loss to follow-up: 52 | 6 months | Group 1: E-learning  Group 2: Group training  Group 3: Basic short training  Groups 1 and 2 were trained using an online self-management portal by Portavita. | 1. TTR  There was no significant difference between the three groups  2. Adverse events  There was no significant difference between the three groups | There were no differences in therapeutic control and usage of the eHealth platform between anticoagulation self-management patients trained by e-learning and by group training. |
| Cao et al.,  2018; China | Cohort study | To compare monitoring outcomes and complications of warfarin therapy managed by pharmacists via hospital or online. | Sample: (N=152)  Mean age: 59.1 years  Male: 83 Female:69  Valve type: mechanical valve Loss to follow-up: - | 12 months | 1. Intervention group: online anticoagulation clinic.  2. Control group: hospital anticoagulation clinics. | 1. TTR  Patients managed in the HAC were more stable INR values within the target therapeutic range (80.2 vs. 71.9%, P=0.005).  2. Adverse events  No significant differences. | The management of OA therapy online yielded similar clinical outcomes compared to that achieved by management via the hospital, although the incidence of supra-therapeutic INR values was increased. |
| Brasen et al.,  2019; Denmark | RCT | To investigate the use of criteria-driven healthcare interactions. | Sample: (N=87)  Mean age: 69.4 years  Male: 69 Female:18  Valve type: mechanical valve  Loss to follow-up: 3 | 10 months | 1. Intervention group: patient self-management and remote monitoring.  2. Control group: patient self-testing INR. | 1. Interactions  The patient self-testing group was handled using 4.2 per month, the PSM group used 1.1 per month.  2. INR  No differences were seen in average INR and TTR in the two groups or the start compared with the end. | Using criteria to guide patient  self-management interactions maintain a good treatment effect while reducing healthcare expenses. |
| Zhu et al.,  2021; China | RCT | To evaluate the safety and efficacy of a anticoagulation management model based on internet technology compared with the traditional outpatient anticoagulation management model for patients after heart valve replacement. | Sample: (N=721)  Age: 18 to 65 years  Male: 440 Female:281  Valve type: mechanical valve  Loss to follow-up: - | 12 months | 1. Intervention group: Internet-based warfarin management.  2. Control group:  Routine AM after discharge from the hospital. | 1. TTR  the internet-based group showed a significantly higher TTR (mean 0.53 vs mean 0.46; P<0.001).  2. Adverse events  The bleeding and embolic events had a lower frequency in the internet-based group (6.94% vs 12.74%; P=0.01). | Internet-based warfarin management is superior to conventional management, which can reduce anticoagulant complications in patients receiving long-term warfarin anticoagulant therapy. |
| Jiang et al.,  2021; China | Cohort study | To evaluate the effect of a pharmacist-led remote warfarin management model using a smartphone application on anticoagulation therapy. | Sample: (N=100)  Age (median): 50.5 years  Male: 55 Female:45  Valve type: mechanical valve  Loss to follow-up: - | 6 months | 1. Intervention group: offered a pharmacist-led remote warfarin management model using the application.  2. Control group: received routine oral medication education by pharmacists. | 1. Fraction of TTR  The median value of the fraction of TTR was 80.3% and 72.1% in the intervention and control groups respectively (P<0.033).  2. Adverse events:  No significant differences. | Remote warfarin management by pharmacists using Yixing improved patients' awareness of warfarin treatment and the fraction of TTR. |
| Cao et al.,  2021; China | Cohort study | To explore the effectiveness and safety of warfarin management via the Alfalfa app, so as to provide  evidence in support of anticoagulant management through online services. | Sample: (N=824)  Age (median):  Alfalfa group: 51.4 years  Offline group: 53.0 years  Male: 412 Female: 412  Valve type: mechanical valve  Loss to follow-up: - | - | 1. Alfalfa group: Patients reported warfarin dosage, INR and related data via the app, with healthcare providers using it to remote warfarin management.  2. Offline group: patients went to the hospital clinic for warfarin management. | 1. TTR  The TTR was significantly higher in the Alfalfa app group (79.35% vs 52.38%, P<.001).  2. Adverse events  The incidences of major bleeding events (0.5% vs 3.0%; P=0.005), emergency department visits (3.1% vs 9.3%; P<0.001), and hospital admissions (0.2% vs 3.0%; P=0.001) were lower in the Alfalfa group. | Warfarin management is superior via the Alfalfa app than via offline services in terms of major bleeding events,  warfarin-related emergency department visits, and hospital admissions. |
| Erba et al.,  2022; Italy | Longitudinal study | To evaluate the quality of anticoagulant control after oral anticoagulants in an anticoagulant clinic and the risk of thrombotic events during anticoagulation in patients with mechanical heart valves. | Sample: (N=3647)  Median age: 25.1 years  Male: 1681 Female:1966  Valve type: mechanical valve  Loss to follow-up: 98 | 13 months | 1. The outpatient OA therapy monitoring program is supported by DDS.  2. Patients can send INR results back to the clinic via "WhatsApp". | 1. Quality of anticoagulant control:  TTR: ≤37 % (n=924)  TTR: 38–52 % (n=893)  TTR: 53–66 % (n=884)  TTR: ≥67 % (n=946)  2. Thrombotic events: (n=70)  3. Thromboembolic complications: (n=77) | Efforts should be made to decrease the number of non-compliant patients and to reach a guideline-recommended TTR of ≥65 %. |
| Alanazi et al.,  2022;  Saudi Arabia | Crossover study | Compare anticoagulation management quality in virtual versus in-person clinics during the COVID-19 pandemic. | Sample: (N=192)  Mean age: 58.6 years  Male:116 Female:76  Valve type: mechanical valve  Loss to follow-up: - | 1-3 months | 1. Intervention group: virtual visit doctors.  2. Control group: actual in-person visits. | 1. TTR:  Median TTR was 54.6% for on-site clinics and 50.0% for virtual clinics (P=0.07). | Virtual clinic results were comparable to in-person clinics for AM during the COVID-19 pandemic. |
| Jiang et al.,  2022; China | Cohort study | To explore the effectiveness, and safety of internet-based warfarin management during the pandemic. | Sample: (N=138)  Mean age: 50.4±13.1 years  Male: 65 Female:73  Valve type: unspecified  Loss to follow-up: 21 | 3 months | 1. Intervention group: online management and used the Alfalfa application.  2. Control group: Offline routine management/ | 1. TTR   TTR was significantly higher in the online group (61.0% vs. 39.6%, P<0.01).   1. Adverse events   Minor bleeding events (28.3% vs. 5.3%, P<0.01) and warfarin-related emergency hospital visits (23.3% vs. 1.8%, P=0.02) were significantly more common in the offline group. | Online management can improve patients’ TTR and reduce anticoagulation-related clinical events. |
| Amruthlal et al.,  2022; India | Cross-sectional study | To develop a simple user-friendly Android mobile application for patients to use the algorithm to predict the dosage of vitamin K antagonists. | Sample: (N=1100)  Age: Mainly over 40 years  Gender: Not reported  Valve type: mechanical valve  Loss to follow-up: - | - | Patients simply enter their INR value into the application to predict their warfarin dose for the next week. | In a practice involving 1092 patients, there was no significant difference between anticoagulant doses derived from the application and those derived from a cardiac specialist. | Remote patients with stable INR values can use the application to predict anticoagulant doses and avoid frequent doctor visits. |

^a^RCT: randomized controlled trial; ^b^OA: oral anticoagulants; ^c^TTR: time in therapy range; ^d^INR: international normalized ratio; ^e^DSS: decision support systems; ^f^AM: anticoagulation management; ^g^AC: Anticoagulant clinic; ^h^TIR: Target INR Range.
